# Maize and peanut intercropping improves the nitrogen accumulation and yield per plant of maize by promoting the secretion of flavonoids and abundance of *Bradyrhizobium* in rhizosphere

**DOI:** 10.3389/fpls.2022.957336

**Published:** 2022-08-04

**Authors:** Qiqi Dong, Xinhua Zhao, Dongying Zhou, Zhenhua Liu, Xiaolong Shi, Yang Yuan, Peiyan Jia, Yingyan Liu, Penghao Song, Xiaoguang Wang, Chunji Jiang, Xibo Liu, He Zhang, Chao Zhong, Feng Guo, Shubo Wan, Haiqiu Yu, Zheng Zhang

**Affiliations:** ^1^College of Agronomy, Shenyang Agricultural University, Shenyang, Liaoning, China; ^2^Shandong Academy of Agricultural Sciences, Jinan, Shandong, China

**Keywords:** root interaction, yield per plant, nitrogen accumulation, rhizosphere soil enzymes, flavonoids, *Bradyrhizobium*

## Abstract

Belowground interactions mediated by root exudates are critical for the productivity and efficiency of intercropping systems. Herein, we investigated the process of microbial community assembly in maize, peanuts, and shared rhizosphere soil as well as their regulatory mechanisms on root exudates under different planting patterns by combining metabolomic and metagenomic analyses. The results showed that the yield of intercropped maize increased significantly by 21.05% (2020) and 52.81% (2021), while the yield of intercropped peanut significantly decreased by 39.51% (2020) and 32.58% (2021). The nitrogen accumulation was significantly higher in the roots of the intercropped maize than in those of sole maize at 120 days after sowing, it increased by 129.16% (2020) and 151.93% (2021), respectively. The stems and leaves of intercropped peanut significantly decreased by 5.13 and 22.23% (2020) and 14.45 and 24.54% (2021), respectively. The root interaction had a significant effect on the content of ammonium nitrogen (NH_4_^+^-N) as well as the activities of urease (UE), nitrate reductase (NR), protease (Pro), and dehydrogenase (DHO) in the rhizosphere soil. A combined network analysis showed that the content of NH_4_^+^-N as well as the enzyme activities of UE, NR and Pro increased in the rhizosphere soil, resulting in cyanidin 3-sambubioside 5-glucoside and cyanidin 3-O-(6-Op-coumaroyl) glucoside-5-O-glucoside; shisonin were significantly up-regulated in the shared soil of intercropped maize and peanut, reshaped the bacterial community composition, and increased the relative abundance of *Bradyrhizobium*. These results indicate that interspecific root interactions improved the soil microenvironment, regulated the absorption and utilization of nitrogen nutrients, and provided a theoretical basis for high yield and sustainable development in the intercropping of maize and peanut.

## Introduction

Intercropping, a diversified planting pattern, can effectively increase farmland ecosystems diversity and maintain sustainable agricultural development (Stomph et al., 2020). The yield advantage of cereal and legume intercropping systems is evident in various intercropping patterns (Raseduzzaman and Jensen, 2017; Zhang et al., 2021). Interspecific interactions are important for improved nutrient utilization and high crop yields in maize and peanut intercropping (Li et al., 2001; Wang et al., 2021; Jiao et al., 2021b). For instance, Jiao et al. (2021b) found that nitrogen accumulation per plant in intercropped peanut decreased significantly (by 25–35%), whereas that in intercropped maize increased significantly at maturity. With the root barrier, the nitrogen accumulation per plant in intercropped maize and intercropped peanut is significantly lower than that without the barrier (Jiao et al., 2021b). It is necessary to understand the critical roles of underground interspecific interactions in nutrient acquisition and productivity in intercropping.

The rhizosphere is a key area for roots to obtain water and nutrients, and it interacts closely with soil physical, chemical, and biological components (Inal et al., 2007). Root-soil interactions, including rhizosphere sensing, root structure and function, and root-induced rhizosphere processes, are critical for soil health, sustainable food security, and resource use efficiency (Wang et al., 2020b). Chemicals secreted by roots into the soil may provide carbon to bacteria or have the potential to act as signaling molecules in interspecific interactions to improve the soil environment, thereby affecting nutrient availability, diffusion, and mass flow (Lian et al., 2002; Zhang et al., 2016; Liu et al., 2022a). Recent studies have reported that maize root exudates can promote interspecific reciprocity to enhance nitrogen fixation in intercropped legume crops (Jiang et al., 2022; Liu et al., 2022a). The root exudates of maize promote the synthesis of flavonoids in faba bean, increase nodulation, and simultaneously enhance gene expression, which subsequently stimulates nitrogen fixation. For example, the genes *NODL4*, *ENODL2*, and chalcone-flavonoid isomerase (*CFI*) involved in flavonoid synthesis are up-regulated (Hu et al., 2021), and the expression of key nodulation genes such as *NOD* is up-regulated (Li et al., 2016a). Flavonoids are key signaling substances in legume nodulation (Hassan and Mathesius, 2012; Leoni et al., 2021), therefore, it is worth exploring how they communicate with neighboring plants in intercropping systems.

Soil micro-food webs play important roles in regulating soil nutrients and plant growth performance to varying degrees (Liu et al., 2022b). Therefore, many studies have been conducted on the soil microbiome related to the intercropping of cereals and legumes (Inal et al., 2007; Zhang et al., 2020b; Pang et al., 2021). A previous study found that the relative abundance of beneficial bacteria, such as *RB41*, *Candidatus-Udaeobacter*, *Stropharia*, *Fusarium* and *Penicillium* increased in the rhizosphere soil under the intercropping of maize and peanut (Zhao et al., 2022a). Studies have also shown that more than half of the increase in nitrogen fixation in intercropped broad beans is due to key bacteria, such as, *Agromyces*, *Arthrobacter*, *Bacillus*, *Lysobacter*, *Paenibacillus*, *Gemmatimonas*, *Heliobacillus*, *Natronocella*, and *Sorangium* recruited by maize root exudates play an important role in root interactions (Hu et al., 2021). In addition, plants produce secondary metabolites and interspecific interactions affect the growth of specific microorganisms in the rhizosphere (Wang et al., 2022). For example, the concentration of flavonoids increases in root exudates and is enhanced between AMF and *Triadica sebifera* (Tian et al., 2021). Neighboring cassava stimulates ethylene release in peanut roots, increases the abundance of Actinobacteria, and reshapes rhizosphere microbial composition (Chen et al., 2020). However, the effect of crop interspecies interactions on the composition of rhizosphere microbial communities is highly complex and dynamic. Hence, it is important to explore the regulatory mechanism of flavonoids on components of soil micro-food webs in the intercropping of maize and peanut, and to provide a theoretical basis to protect the health and sustainable development of soil ecosystems.

In this study we elucidate how root exudates assemble rhizosphere microbes and the interactions between root exudates and rhizosphere microbes. To this end, we combined soil metabolomics with metagenomic sequencing technology to study the interaction of root exudates–soil microorganisms–soil and the influence of nitrogen uptake, transport, and yield of intercropping systems under different root separation modes of maize and peanut intercropping. Therefore, this study aimed to (1) reveal the effects of maize and peanut root interactions on soil nutrient cycling and soil enzyme activities, (2) explore the relationship between flavonoids root exudates and soil bacterial community composition, and (3) clarify the correlation of soil nutrients and enzymatic activities, flavonoids root exudates, and rhizosphere soil bacteria, and to promote the yield of common driving mechanisms in intercropping systems.

## Materials and methods

### Experimental setup

**Figure 1:**
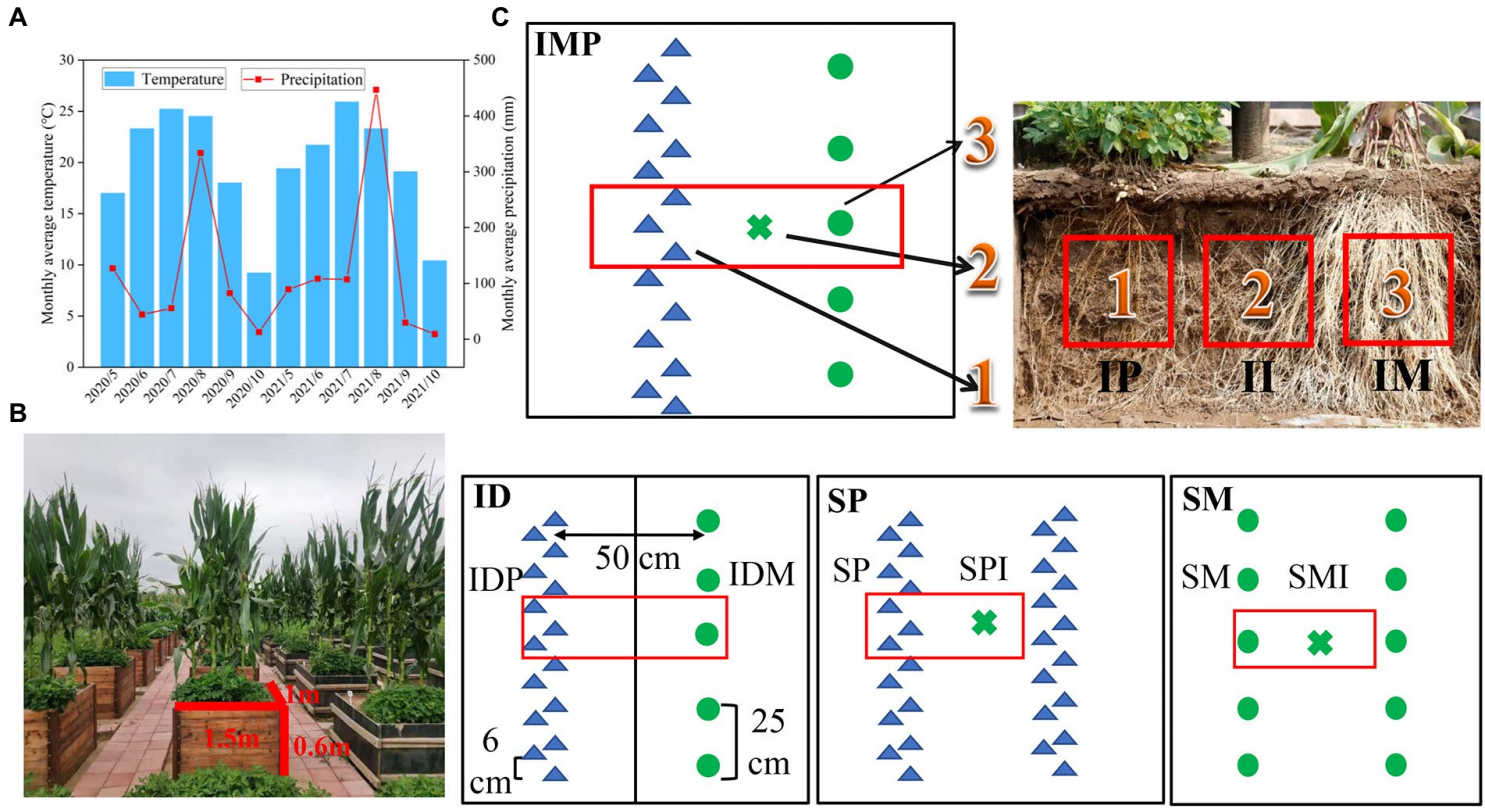
Overview of the root box simulation experiment. **(A)** The average monthly temperature and precipitation in the 2020 and 2021 growing seasons. **(B)** The distribution of root box simulation experiment. **(C)** Diagram of plant and soil samples in different root interaction patterns. ●: maize plants, ▲: peanut plants, □: plant and rhizosphere soil sample area, ×: the shared soil sample. IP: intercropped peanut, II: the shared soil of intercropped maize and peanut, IM: intercropped maize, IDP: intercropped peanut with board separation, IDM: intercropped maize with board separation, SP: sole peanut, SPI: the shared soil of sole peanut, SM: sole maize, SMI: the shared soil of sole maize.

This experiment was conducted in the experimental field of Shenyang Agricultural University, the Northeast Region Crop Cultivation Scientific Observatory of the Ministry of Agriculture and Rural Affairs from 2020 to 2021 (41°82′ N, 123°56′ E). The experimental site has a typical semi-arid continental and monsoon climate, with the average monthly temperature and precipitation shown in Figure 1A. The soil was classified as brown loam. In the top 20 cm of the soil profile, the soil contained organic matter (SOM), alkaline hydrolysable nitrogen (AN), available phosphorus (AP), available potassium (AK), and pH were 14.59 g kg^−1^, 178.07 mg kg^−1^, 43.82 mg kg^−1^, 201.86 mg kg^−1^, and 6.5, respectively, in 2020. The soil contained 14.85 g kg^−1^, 199.64 mg kg^−1^, 56.87 mg kg^−1^, and 213.36 mg kg^−1^ of SOM, AN, AP, and AK, respectively, and its pH was 6.5 in 2021.

### Experimental design

This experiment included the following four planting patterns: (1) intercropping of maize and peanut (IMP); (2) intercropping of maize and peanut with board separation (ID), ensuring that the maize and the peanut roots were not in contact; (3) sole maize; and (4) sole peanut, there were three replicates per planting pattern (Figure 1B). Two rows of crops were placed in each root box at a row distance of 50 cm. The intercropping planting patterns were one row of maize and one row of peanuts, and the interplant distances for intercropped maize and peanut were 25 cm and 6 cm, respectively. Both sole maize and sole peanut had two rows, and the interplant distance was the same as that in intercropping (Figure 1C). The maize used was Liang-yu 99 (*Zea mays* L.) from Dandong Denghai Seed Industry Co. Ltd., China. The peanut used was Nong-hua 9 (*Arachis hypogaea* L.) from the Peanut Research Institute of Shenyang Agricultural University, China. The maize and peanut were sowed on 15 May, 2020 and 18 May, 2021, respectively, and harvested on15 September, 2020, and 18 September, 2021, respectively. In all treatments, the topsoil (0–25 cm depth) was ploughed every year before cultivation. The amount of fertiliser applied per planting pattern is listed in Supplementary Table S1. Other cultivation and management measures were the same as those used for the local production.

One maize plant and three peanut plants were randomly collected in each planting pattern for nitrogen accumulation determination at 60 days and 120 days after sowing, as follows: intercropped maize (IM), intercropped peanut (IP), intercropped maize with board separation (IDM), intercropped peanut with board separation (IDP), sole maize (SM), and sole peanut (SP), the collections were repeated three times (Figure 1C). Rhizosphere soil samples were collected for soil nitrogen content and enzyme activity as follows: IM, IP, the shared soil of intercropped maize and peanut (II), IDM, IDP, SM, the shared soil of sole maize (SMI), SP, and the shared soil of sole peanut (SPI), the collections were repeated three times (Figure 1C). To explore the differences in root exudates and microbial community under the different planting patterns (including SM, SP, and IMP), a part of the rhizosphere soil samples was collected at 60 days after sowing in 2020, stored in an ice box, transported to the laboratory, and stored at −80°C for sequencing analysis.

### Determination of yield per plant and components

Three maize plants and five peanut plants were selected for harvest in each planting pattern after sowing for 120 days, the collections were repeated three times and the plants were air-dried to a constant weight. The yield per plant, ear length, ear coarse, and spike grain number of the maize were measured. The yield per plant, pods per plant, full pods per plant, 100-pod weight, and 100-kernel weight of peanuts were measured.

### Nitrogen accumulation in various organs of the plant

The plant samples were divided into roots, stems, leaves, and pods (grains). Then, they were placed in an oven at 105°C for 30 min and dried at 80°C to a constant weight. After dry matter determination, the samples of each organ of the plant were crushed through a 0.5-mm sieve for plant nitrogen determination. The total nitrogen accumulation was determined using the Kjeldahl method (FOSS, Denmark, Kjeltec 8,400; Li et al., 2001).

### Rhizosphere soil nitrogen content and enzyme activity

Rhizosphere soil samples were collected in the same way as described in a (Zhao et al., 2022a). Total nitrogen (TN) was measured using the Kjeldahl method, and the soil ammonium nitrogen (NH_4_^+^-N) was measured using the colourimetric method of Nessler’s reagent.

Rhizosphere soil samples collected at 60 days after sowing were tested for soil enzyme activity. The activities of the soil enzymes urease (UE), nitrate reductase (NR), protease (Pro), and dehydrogenase (DHO) were measured using an ELISA kit (MLBIO, Shanghai, China; Zhao et al., 2022a).

### Root exudate extraction

The samples were then thawed on ice. We homogenized 50 mg of one sample with 500 μl ice-cold methanol/water (70%, v/v). The mixture was then homogenized at 30 Hz for 2 min. After homogenisation, the mixture was shaken for 5 min and incubated on ice for 15 min. The mixture was centrifuged at 12,000 rpm at 4°C for 10 min, and 400 μl of the supernatant was placed into another centrifuge tube. Next, 500 μl of ethyl acetate/methanol (V, 1:3) was added to the original centrifuge tube, the mixture was oscillated for 5 min, and then incubated on ice for 15 min. Then, the mixture was centrifuged at 12,000 rpm at 4°C for 10 min. We took 400 μl of the supernatant and combined and concentrated the two supernatants. Next, we added 100 μl of 70% methanol water to the dried product, and ultrasonic treatment was performed for 3 min. Finally, the mixture was centrifuged at 12,000 rpm at 4°C for 3 min, and 60 μl of the supernatant was extracted for LC–MS/MS analysis. Root exudates were detected using an ultra-high-performance liquid chromatograph (Agilent 7,890, Santa Clara, CA, United States) and a mass spectrometer (QTOF/MS-6545, LECO, St. Joseph, MI, United States) for sequencing at Metware, Wuhan, China.

### Quantification of flavonoids and isoflavonoids in root exudates by LC–MS/MS

All samples were acquired using an LC–MS system followed by machine ordering. The analytical conditions were as follows:

Ultra Performance Liquid Chcromatography (UPLC): Column, Waters ACQUITY UPLC HSS T3 C18 (1.8 μm, 2.1 mm × 100 mm); column temperature, 35°C; flow rate, 0.3 ml/min; injection volume, 1 μl; solvent system, water (0.01% methanolic acid): acetonitrile; gradient programme of positive ion, 95:5 V/V at 0 min, 79:21 V/V at 3.0 min, 50:50 V/V at 5.0 min, 30:70 V/V at 9.0 min, 5:95 V/V at 10.0 min, and 95:5 V/V at 14.0 min; gradient programme of negative ion, 95:5 V/V at 0 min, 79:21 V/V at 3.0 min, 50:50 V/V at 5.0 min, 30:70 V/V at 9.0 min, 5:95 V/V at 10.0 min, and 95:5 V/V at 14.0 min. The original data file obtained by LC–MS analysis was first converted into the mzML format using ProteoWizard software. Peak extraction, alignment, and retention time correction were performed using the XCMS program. The SVR method was used to correct the peak area. The peaks with a deletion rate of > 50% in each group of samples were filtered. Subsequently, metabolic identification information was obtained by searching the laboratory’s self-built database and integrating the public database with met DNA.

### Assessment of microbial community structure in rhizosphere soils by metagenomic sequencing

Total DNA was extracted from rhizosphere soil samples (0.5 g) using the Qubit® dsDNA Assay Kit in a Qubit® 2.0 Flurometre (Life Technologies, CA, United States) following the manufacturer’s instructions. DNA concentration and purity were estimated using a Nanodrop 1,000 spectrophotometre (Thermo Fisher Scientific, Waltham, MA, United States) and electrophoresis on 1% (w/v) agarose gel. The OD value was between 1.8 and 2.0 and DNA contents higher than 1 μg were used to construct the library. The detailed determination of metagenomic sequencing results is shown in Supplementary Data Text S1. Sequence data were submitted to the Sequence Read Archive (PRJNA833565; https://www.ncbi.nlm.nih.gov/sra/PRJNA833565).

### Statistical analysis

The yield per plant and components, nitrogen accumulation, soil nutrient content, and soil enzyme activity were assessed by one-way analysis of variance (ANOVA) with Duncan’s test using SPSS 23.0 (IBM SPSS Inc., United States). Differences were considered statistically significant at p < 0.05. The figures were plotted using Origin Pro (version 9.0; Origin Lab Corporation, Northampton, MA, United States).

Quality control (QC) samples were prepared by mixing sample extracts and were used to analyze the repeatability of samples using the same processing method. During instrumental analysis, a quality control sample was inserted into every 15 assay samples to monitor the repeatability of the analytic process. Principal component analysis (PCA) was performed to probe the changes in root exudates and microbial community with different planting patterns, respectively. PCA was conducted using the base package of R (Version 3.5.0). Orthogonal partial least squares discriminant analysis (OPLS-DA) was a multivariate statistical analysis method with supervised pattern recognition, which could effectively eliminate irrelevant effects to screen differential root exudates. OPLS-DA analysis was conducted using the MetaboAnalystR package. DIAMOND (Buchfink et al., 2015) software (V0.9.9, https://github.com/bbuchfink/diamond/) to blast the Unigenes to the bacterial sequences, which were all extracted from the NR database (Version: 2018-01-02, https://www.ncbi.nlm.nih.gov/) of NCBI with the parameter settings blastp, −e 1e-5. LEfSe analysis was conducted using the LEfSe software (the default LDA score was 3; Segata et al., 2011). To clarify the correlation between root exudates, rhizosphere soil microorganisms, and soil physicochemical properties, a network heat map was drawn using https://www.omicshare.com. Including Spearman’s correlation analysis of root exudates and dominant bacteria, and Mantel test correlation analysis of soil physicochemical properties and root exudates and rhizosphere soil microorganisms (Zhu et al., 2019). Random forest modelling was conducted to quantitatively assess the important predictors of plant yield (Chen et al., 2020), including nitrogen accumulation per plant of maize and peanut, soil nutrients (TN: soil total N, NH_4_^+^-N: soil ammonium N), soil enzyme activity (UE: soil urease; NR: soil nitrate reductase; Pro: soil protease; DHO: soil dehydrogenase), core root exudates in the shared soil of intercropped maize and peanut, and *Bradyrhizobium*. These analyses were conducted using the randomForest package, and the significance of the model and predictor importance were determined using the rfUtilities and rfPermute packages in R software, respectively.

## Results

### Responses of maize and peanut yield per plant under different planting patterns

Planting patterns and year × planting patterns significantly affected (*p* < 0.05) the maize and peanut yield per plant (Table 1). Compared to SM, IDM significantly decreased by 58.12% (2020, *p* < 0.05) and 32.50% (2021), and IM significantly increased by 21.05% (2020, *p* < 0.05) and 52.81% (2021, *p* < 0.05). Compared to SP, IDP decreased by 6.14% (2020), and significantly increased by 27.57% (2021, *p* < 0.05), and IP significantly decreased by 39.51% (2020, *p* < 0.05) and 32.58% (2021, *p* < 0.05). The pods per plant and 100-kernel weight significantly affected peanut yield.

**Table 1:** Yield per plant and components of maize and peanut under different planting patterns.

Year	Planting patterns	Maize				Peanut					Yield per plant/g	Ear Length/cm	Ear coarse/cm	Spike grain number	Yield per plant/g	Pods per plant	Full pods per plant	100-pod weight/g	100-kernel weight/ g
2020	SM/SP	485.56 ± 50.21b	17.22 ± 1.25a	16.28 ± 1.09a	605.28 ± 60.08a	30.93 ± 2.06a	24.40 ± 0.33b	22.47 ± 0.68b	156.88 ± 18.96a	67.42 ± 5.05ab
	ID	203.33 ± 5.44c	15.29 ± 2.40a	15.12 ± 0.51b	505.44 ± 100.31a	29.03 ± 2.62a	31.87 ± 2.89a	29.13 ± 3.83a	170.83 ± 12.84a	60.95 ± 2.49b
	IMP	587.78 ± 29.36a	17.39 ± 2.35a	16.08 ± 0.83a	570.00 ± 132.45a	18.71 ± 0.59b	19.60 ± 0.33c	16.53 ± 0.93c	142.45 ± 3.43a	69.92 ± 1.11a
2021	SM/SP	355.56 ± 17.50b	16.67 ± 0.25a	13.37 ± 0.81a	544.00 ± 127.07b	29.31 ± 2.12b	24.53 ± 0.25b	18.40 ± 3.82b	154.18 ± 22.55a	66.53 ± 6.75a
	ID	240.00 ± 46.43b	18.07 ± 0.74a	15.80 ± 1.48a	567.17 ± 20.37a	37.39 ± 3.23a	35.47 ± 1.75a	29.07 ± 1.18a	172.37 ± 13.53a	66.40 ± 6.46a
	IMP	543.33 ± 66.22a	17.50 ± 2.16a	16.00 ± 1.56a	665.67 ± 73.38ab	19.76 ± 1.42c	18.27 ± 0.66c	11.40 ± 4.70b	163.18 ± 32.93a	69.75 ± 9.21a
Year		0.078				0.061				
Planting patterns	0.000				0.000				
Year×Planting patterns	0.045				0.019				

Different lowercase letters represent significant difference level *p* = 0.05 using Duncan’s test, as follows. SM: sole maize, SP: sole peanut, ID: intercropping of maize and peanut with board separation, IDM: intercropped maize with board separation, IDP: intercropped peanut with board separation, IMP: intercropping of maize and peanut, IM: intercropped maize, IP: intercropped peanut.

### Responses of nitrogen accumulation in various organs of maize and peanut under different planting patterns

Compared with SM, the nitrogen accumulation per plant of IDM and IM increased (Supplementary Figures 1A,B), with the significantly increased in IM. The year, planting patterns, and year × planting patterns significantly affected nitrogen accumulation in maize roots, stems, and leaves, and the planting patterns significantly affected nitrogen accumulation in grains (Supplementary Table S2). Nitrogen accumulation in various organ of IDM and IM increased. Among them, the nitrogen accumulation of IM roots significantly increased by 129.16% (2020, *p* < 0.05) and 151.93% (2021, *p* < 0.05). Compared with SP, the nitrogen accumulation per plant of IDP and IP decreased significantly, with the highest decrease in IP at 120 days (Supplementary Figures 1C,D). The year, planting patterns, and year × planting patterns significantly affected nitrogen accumulation in the roots and leaves of peanuts (Supplementary Table S2). The nitrogen content of stems and leaves in IP decreased by 5.13 and 22.23% (2020) and 14.45 and 24.54% (2021), respectively. The nitrogen accumulation in the roots, leaves, and pods of peanuts decreased significantly at 120 days after sowing (Supplementary Table S2).

### Responses of rhizosphere soil nitrogen content and enzyme activity of maize and peanut under different planting patterns

The year significantly affected the TN and NH_4_^+^-N contents at 120 days (Supplementary Table S3). Compared with SM, the TN and NH_4_^+^-N contents increased in IDM and IM, and the increase was highest in IM at 60 days. At 120 days, the NH_4_^+^-N content increased significantly in the IM. Compared with the SP, the TN and NH_4_^+^-N contents increased in IDP and IP, and the NH_4_^+^-N content increased significantly in IP at 60 days and 120 days, respectively. Compared with SMI and SPI, the TN and NH_4_^+^-N contents (*p* < 0.05) increased in II at 60 days and 120 days, respectively. Thus, root interaction had a significant effect on the NH_4_^+^-N content in the rhizosphere soil.

Compared with SM, the activities of UE, Pro, NR, and DHO increased in IDM; UE and Pro increased significantly by 33 and 17% (2020) and 6 and 10% (2021), respectively (Supplementary Figures 2A,B). The activities of UE, Pro, and DHO increased in IM; UE and Pro increased significantly by 36 and 28% (2020) and 4 and 30% (2021), respectively (Supplementary Figures 2A–C). Compared with SP, the activities of UE, Pro, and DHO in IDP increased. The NR activity in IDP decreased significantly by 2% (2021). The activities of UE, Pro, NR, and DHO in IP increased; Pro and NR increased significantly by 31 and 56% (2020) and 32 and 44% (2021), respectively (Supplementary Figures 2B,C). The activities of UE, Pro, and DHO in II were higher than those of SMI and SPI (Supplementary Figures 2A,B,D). The NR activity was lower than those of SMI and SPI (Supplementary Figure 2C). Thus, the root interaction had a positive effect on the enzyme activities of UE, Pro, NR, and DHO in the rhizosphere soil of intercropped maize, intercropped peanuts, and the shared soil of intercropped maize and peanuts.

### Responses of the root exudates of maize and peanut under different planting patterns

**Figure 2:**
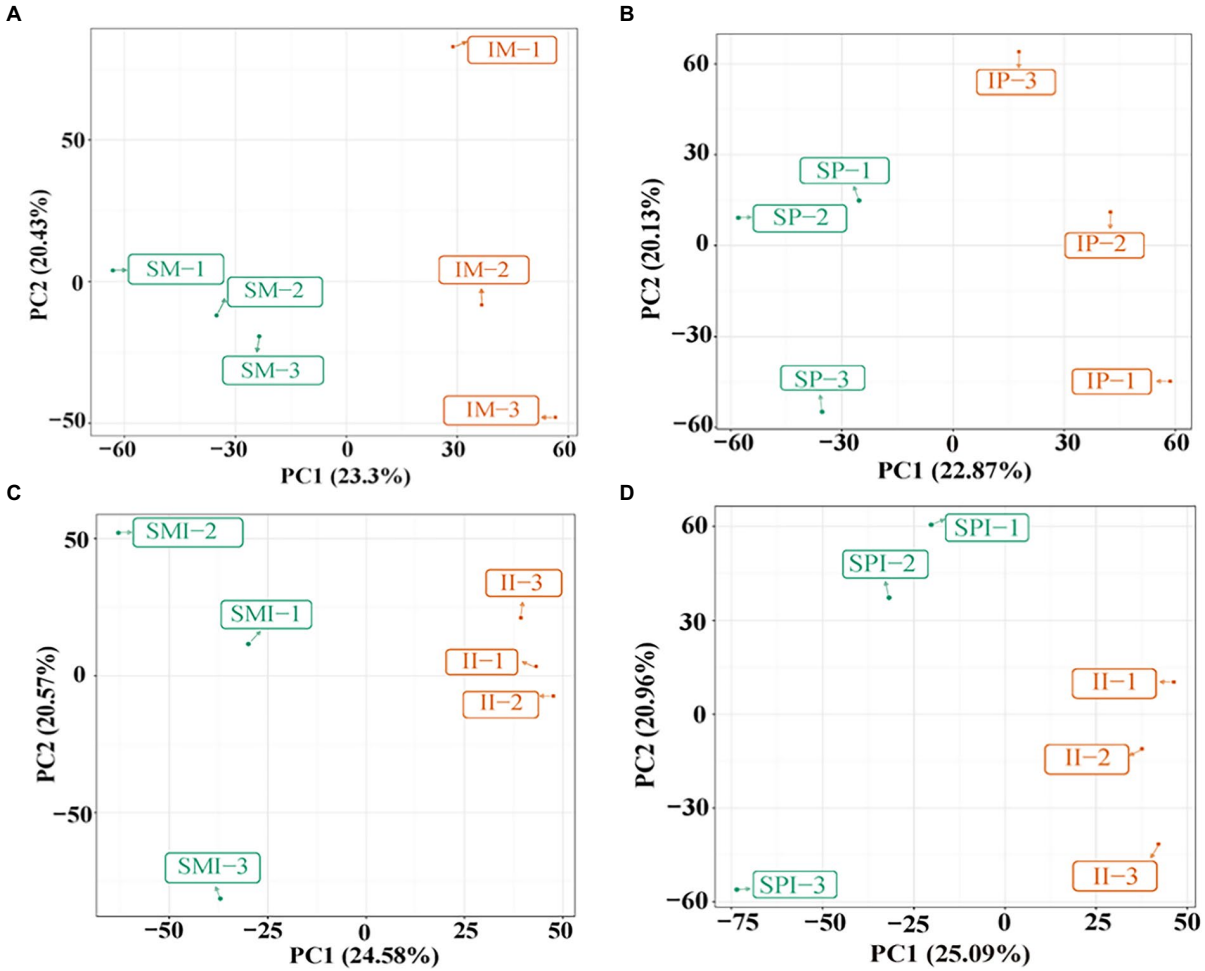
Principal component analysis (PCA) of maize and peanut root exudates under different planting patterns. **(A)** SM vs. IM, **(B)** SP vs. IP, **(C)** SMI vs. II, **(D)** SPI vs. II. SM: sole maize, IM: intercropped maize, SP: sole peanut, IP: intercropped peanut, SMI: the shared soil of sole maize, SPI: the shared soil of sole peanut, II: the shared soil of intercropped maize and peanut.

**Figure 3:**
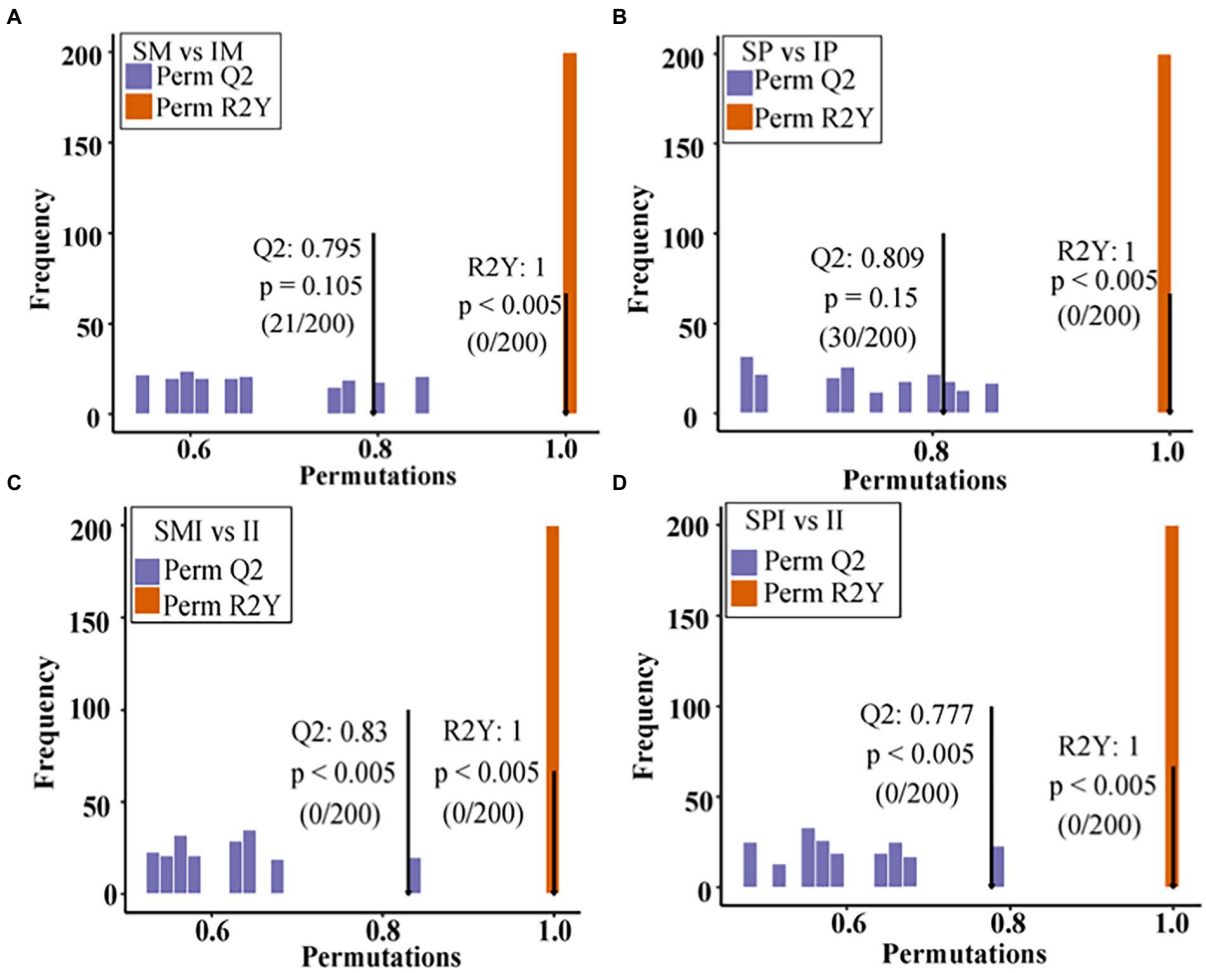
OPLS-DA models of maize and peanut root exudates under different planting patterns. **(A)** SM vs. IM, **(B)** SP vs. IP, **(C)** SMI vs. II, **(D)** SPI vs. II. X: the accuracy of the model, Y: the frequency of the model classification effect. SM: sole maize, IM: intercropped maize, SP: sole peanut, IP: intercropped peanut, SMI: the shared soil of sole maize, SPI: the shared soil of sole peanut, II: the shared soil of intercropped maize and peanut.

The PCA of the samples (including the QC samples) showed that the mixed samples of the treatments were clustered together, indicating that the sequencing results were stable and reliable, and the samples within the group had good repeatability (Supplementary Figure 3). Sixteen root exudate types were detected, of which lipids and lipid molecules (20.42%), organic heterocyclic compounds (15.92%), benzenoids (14.94%), organic oxygen compounds (8.63%), organic acids and derivatives (8.11%), and phenylpropanoids and polyketides (5.93%) were the most abundant (Table 2). The root exudates in SM vs. IM and SP vs. IP were clearly separated along the first principal component, indicating that planting patterns had a significant effect on root exudates (Figures 2A,B). The root exudates of SMI vs. II and SPI vs. II were also significantly separated along the first principal component, suggesting that root interaction significantly affected the distribution of root exudates (Figures 2C,D). The OPLS-DA results showed that the model could better explain the differences between planting patterns (R2Y = 1, Q2 > 0.5; Figure 3), proving that the results of differential root exudates caused by intercropping were stable and reliable.

**Table 2:** The categories of maize and peanut root exudates in rhizosphere soil under different planting patterns.

No.	Root exduates	SM vs IM		SP vs IP		SMI vs II		SPI vs II		%
		Up	Down	Up	Down	Up	Down	Up	Down	
1	Alkaloids and derivatives	1	1	5	1	4	2	3	6	1.73%
2	Benzenoids	30	13	33	18	24	29	34	18	14.94%
3	Homogeneous non-metal compounds	1	1	1	0	0	1	0	1	0.38%
4	Hydrocarbon derivatives	0	0	0	0	1	0	1	0	0.15%
5	Hydrocarbons	0	0	0	0	0	0	1	1	0.15%
6	Lignans, neolignans and related compounds	2	1	0	0	2	0	2	2	0.68%
7	Lipids and lipid-like molecules	31	32	36	24	25	38	42	44	20.42%
8	Nucleosides, nucleotides, and analogues	4	1	4	0	2	1	5	3	1.50%
9	Organic acids and derivatives	14	7	23	10	9	20	12	13	8.11%
10	Organic nitrogen compounds	2	5	5	8	3	7	3	4	2.78%
11	Organic oxygen compounds	13	5	24	10	10	19	21	13	8.63%
12	Organohalogen compounds	0	0	3	1	2	0	0	1	0.53%
13	Organoheterocyclic compounds	22	20	31	24	19	26	41	29	15.92%
14	Organometallic compounds	1	0	2	0	1	0	1	0	0.38%
15	Organosulfur compounds	2	2	1	0	1	5	1	0	0.90%
16	Phenylpropanoids and polyketides	9	8	7	3	14	12	17	9	5.93%
17	–	35	23	23	15	23	32	46	28	16.89%

SM: sole maize, IM: intercropped maize, SP: sole peanut, IP: intercropped peanut, SMI: the shared soil of sole maize, SPI: the shared soil of sole peanut, II: the shared soil of intercropped maize and peanut.

### The response of flavonoids and isoflavonoids in root exudates to the intercropping of maize and peanut

**Figure 4:**
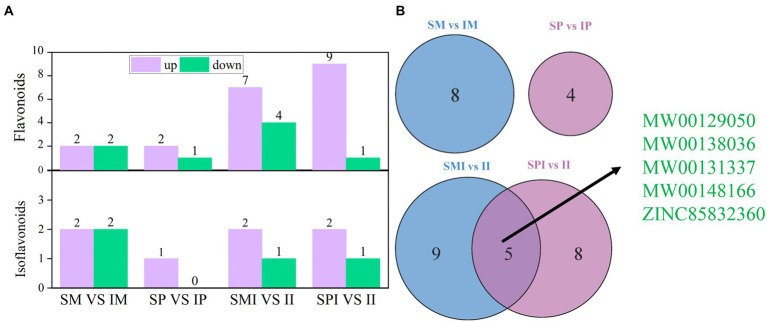
Distribution of flavonoids and isoflavonoids in rhizosphere soils of maize and peanut in different planting patterns. **(A)** The number of flavonoids and isoflavonoids. **(B)** Venn diagram of different planting patterns. SM: sole maize, IM: intercropped maize, SP: sole peanut, IP: intercropped peanut, SMI: the shared soil of sole maize, SPI: the shared soil of sole peanut, II: the shared soil of intercropped maize and peanut.

A total of 23 flavonoids and 7 isoflavonoids were detected in the differential root exudates (Figure 4A and Supplementary Table S4). In addition, the qualitative analysis of flavonoids and isoflavonoid compounds in the differential root exudates detected those four differential flavonoid compounds were annotated in SM vs. IM, with two being upregulated and two being downregulated (Figure 4A). The greatest increase and decrease in root exudates were observed for MW00138036 and MW00153631, respectively (Supplementary Table S4). Four differential isoflavonoid compounds were annotated in SM vs. IM, with two being upregulated and two being down-regulated (Figure 4A). The greatest increase and decrease in root exudates were observed for MW00132749 and MW00162520, respectively (Supplementary Table S4). A total of three differential flavonoid compounds were annotated in SP vs. IP, with two being upregulated and one being downregulated (Figure 4A). The root exudates with the highest increase and decrease were MW00148166 and ZINC38321668, respectively (Supplementary Table S4). One differential isoflavonoid compound, MW00131494, was upregulated. In addition, 11 differential flavonoid compounds were annotated in SMI vs. II, of which seven were upregulated and four were down-regulated (Figure 4A). The greatest increase and decrease in root exudates were observed for MW00138036 and MW00129050, respectively (Supplementary Table S4). Three differential isoflavonoid compounds were annotated in SMI vs. II, of which two were upregulated and one was downregulated (Figure 4A). The greatest increase and decrease in root exudates were observed for MW00131337 and MW00162520, respectively (Supplementary Table S4). A total of 10 differentially expressed flavonoids were annotated in SPI vs. II, of which nine were up regulated and one was downregulated (Figure 4A). The root exudates with the highest increase and decrease were MW00138036 and ZINC15271783, respectively (Supplementary Table S4). Three different isoflavonoid compounds were annotated in SPI vs. II, of which two were up regulated and one was downregulated (Figure 4A). The greatest increase and decrease in root exudates were observed for MW00131337 and MW00139133, respectively. Venn diagram showed that unique and shared differences root exudates between SM vs. IM and SP vs. IP, SMI vs. II and SPI vs. II. Only five core root exudates, MW00129050, MW00138036, MW00131337, ZINC85832360, and MW00148166, were shared between SMI vs. II and SPI vs. II (Figure 4B and Supplementary Table S4). We speculate that five core root exudates were the key differential root exudates affected the process of root interaction. In addition, all flavonoids and isoflavones were matched with the KEGG database to obtain the pathway information. The results showed that the differential root exudates were mainly involved in anthocyanin biosynthesis (https://www.genome.jp/kegg-bin/show_pathway?map00942).

### Responses of the bacterial community composition of maize and peanut under different planting patterns

**Figure 5:**
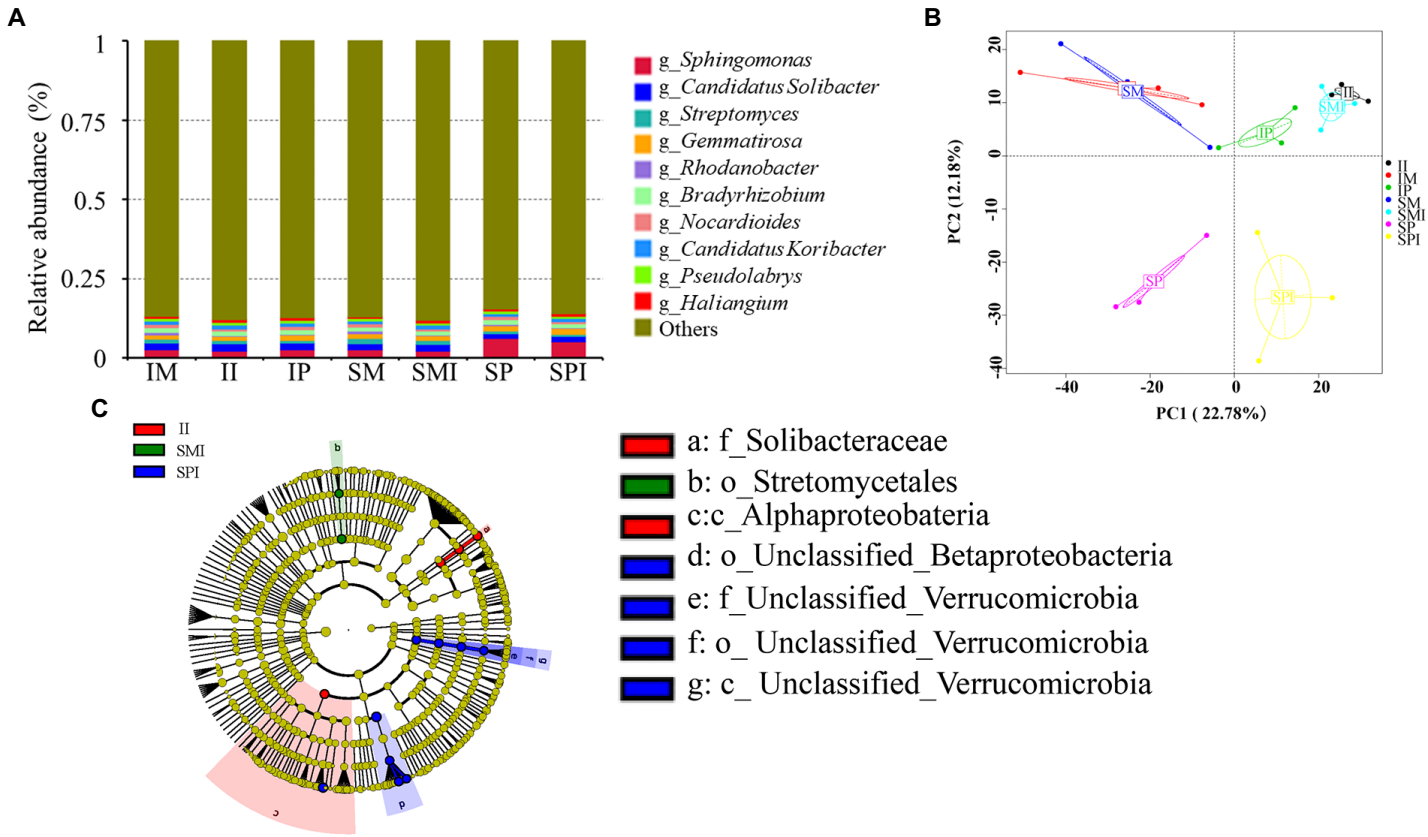
Composition and distribution of bacterial communities at the genus level under different planting patterns. **(A)** Histogram of the relative abundance of bacterial communities. **(B)** Principal component analysis (PCA) of bacterial community. **(C)** LEfse analysis. SM: sole maize, IM: intercropped maize, SP: sole peanut, IP: intercropped peanut, SMI: the shared soil of sole maize, SPI: the shared soil of sole peanut, II: the shared soil of intercropped maize and peanut.

The abundance of unigenes correlation coefficients between different planting patterns was 1, showing that the higher the similarity of gene abundance between samples, the more reliable the experiment and the more reasonable the sample selection (Supplementary Figure 4). According to the unigene annotation, the bacterial taxa *Sphingomonas* (3.30%), *Candidatus Solibacter* (1.96%), *Gemmatirosa* (1.54%), *Bradyrhizobium* (1.39%), *Streptomyces* (1.09%), and *Candidatus Koribacter* (1.01%) were present at high relative abundance (RA; mean RA > 1%; Figure 5A and Supplementary Table S5). Compared with the SM, the relative abundance (RA) of *Bradyrhizobium, Sphingomonas*, and *Candidatus Koribacter* increased by 27.98, 5.49, and 2.43%, respectively in IM. Compared with SP, the RA of *Candidatus Solibacter*, *Candidatus Koribacter*, *Streptomyces*, and *Bradyrhizobium* increased by 39.16, 29.57, 8.93, and 3.59%, respectively. The RA of *Bradyrhizobium*, *Candidatus Solibacter*, and *Candidatus Koribacter* were higher in II than in SMI and SPI. The PCA showed a clear difference in community composition between SP and IP, SPI, and II, and they could be separated along the second coordinate axis (Figure 5B), suggesting that neighbouring maize affects bacterial community composition in IP and II. To further clarify the effect of root interactions on bacterial community composition, LEfSe analysis showed that the abundance of Proteobacteria and Bacteroidetes was significantly higher in II than in SMI and SPI. Within the Proteobacteria phylum, the class Alphaproteobacteria was abundant in II. The family Solibacteraceae, belonging to Bacteroidetes, was also significantly more abundant in II (Figure 5C).

### Correlations among the soil physicochemical properties, root exudates and bacterial community

**Figure 6:**
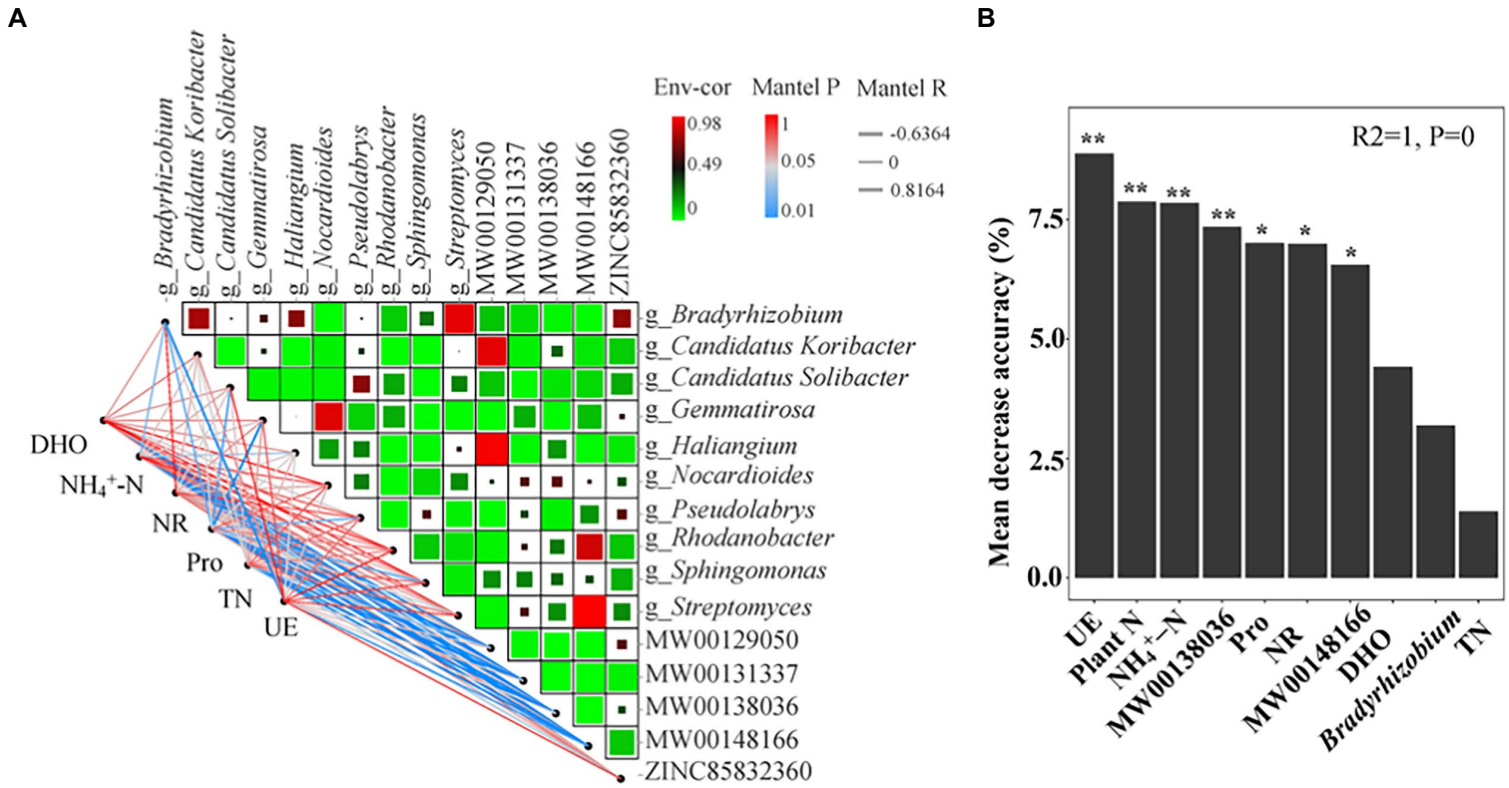
Correlations among the soil physicochemical properties, flavonoids root exudates, and bacterial community. **(A)** A network heatmap of correlations between soil physicochemical properties and root exudates and bacterial communities. **(B)** Mean predictor importance of factors related to plant production based on random forest analysis. The significance levels of each predictor in the random forest analysis are as follows: ^*^p < 0.05 and ^**^p < 0.01. Plant N: nitrogen acumulation per plant in maize and peanut; TN: soil total N; NH_4_^+^–N: soil ammonium N; UE: soil urease; NR: soil nitrate reductase; Pro: soil protease; DHO: soil dehydrogenase; MW00138036: Cyanidin 3-sambubioside 5-glucoside; MW00148166: Cyanidin 3-O-(6-O-p-coumaroyl) glucoside-5-O-glucoside; Shisonin.

Spearman’s correlation analysis reflected the mechanism of root exudates and bacteria under the intercropping of maize and peanut (Figure 6A). The results showed that flavonoids were significantly correlated with bacteria at the genus level, with more positive correlations than negative correlations (Supplementary Table S6). Flavonoids MW00138036 and MW00148166 had the greatest positive correlation with g_*Bradyrhizobium* (*r* = 0.52 and *r* = 0.57, respectively; *p* < 0.05), followed by g_*Haliangium* (*r* = 0.56, *p* < 0.05), g_*Pseudolabrys* (*r* = 0.48, *p* < 0.05), and g_*Candidatus Koribacter* (*r* = 0.44, *p* < 0.05). MW00138036 had a significantly negative correlation with g_*Gemmatirosa* (*r* = −0.58, *p* < 0.05). The isoflavonoid MW00131337 had a significantly positive correlation with g_*Candidatus Solibacter* (*r* = 0.54, *p* < 0.05), g_*Haliangium* (*r* = 0.54, *p* < 0.05), and g_*Candidatus Koribacter* (*r* = 0.47, *p* < 0.05). g_*Bradyrhizobium* had the greatest correlation with flavonoid root exudates as the key flora. This belonged to the same Proteobacteria as the significantly changed flora in II (Figure 5C). The Mantel test correlation analysis showed that Pro, NH_4_^+^-N, and UE were significantly correlated with the bacteria (Figure 6A). Pro had the strongest positive correlation with g_*Candidatus Solibacter* and the strongest negative correlation with g_*Gemmatirosa.* Pro, NH_4_^+^-N, and UE had significantly positive correlations with g_*Bradyrhizobium* (Supplementary Table S7). In addition, TN, Pro, DHO, and NH_4_^+^-N were significantly correlated with root exudates (Supplementary Table S7), indicating that root exudates are key factors affecting changes in soil physicochemical properties and microbial communities. The model indicated that the most important predictor of plant yield was UE, followed by plant N, soil NH_4_^+^-N, MW00138036, Pro, NR, and MW00148166 (*p* < 0.05; Figure 6B). Soil DHO (*p* = 0.06), the RA of *g_Bradyrhizobium* (*p* = 0.13), and soil TN (*p* = 0.25) had no significant influence on plant yield.

## Discussion

The results of this study showed that the yield of intercropped maize was significantly higher than that of sole maize, while the yield of intercropped peanuts was significantly lower than that of sole peanuts (Table 1). These results were consistent with those of previous studies that found that intercropping maize and peanuts was beneficial to maize yield (Zou et al., 2021; Jiao et al., 2021a). The yield advantage of intercropping was that the increase in the intercropped maize yield could offset the decrease in the intercropped peanut yield. In addition to the cultivation patterns, such as planting density (Zhang et al., 2020a) and border-row effect (Wang et al., 2020a), which affect the yield formation of intercropping systems, the internal mechanism changes caused by root interactions between species are worthy of exploration. Nitrogen is one of the main nutrients limiting plant productivity. The biological nitrogen fixation of legume crops can transform and absorb nitrogen in the atmosphere; there is an increasing number of studies on nitrogen uptake and nitrogen transfer in the intercropping of cereals and legumes (Thilakarathna et al., 2016; Luo et al., 2021; Nasar et al., 2022). This study showed that the nitrogen accumulation in each organ of IDM and IM increased. The nitrogen content of the roots, leaves, and pods was significantly lower in peanuts than in sole peanuts (Supplementary Table S2). This is because the dominant crop, maize, competes for higher nutrient uptake when interacting with peanut roots underground (Li et al., 2010). Interspecific competition improves the growth and development of peanut nodules and the synthesis and activity of nitrogenase *nifH*, thereby promoting biological nitrogen fixation in intercropped peanut and nitrogen transfer to neighbouring maize (Carlsson and Huss-Danell, 2013; Chalk et al., 2014; Thilakarathna et al., 2016; Jain et al., 2020; Pang et al., 2021). Studies have shown that the direct or indirect interaction of roots between crops and the characteristics of root ecological separation play a role in interspecific competition, promote nutrient absorption, and improve crop yield (Hochholdinger et al., 2004; Tajima et al., 2008; Jiao et al., 2021a). Thus, root interaction improves the nitrogen absorption and transfer in the intercropping system, showing the interspecific promoting effect, and realizing the high yield of the intercropping system.

The results of this study showed that the nutrient content of the rhizosphere soil was higher in intercropped maize and peanuts than in sole maize and sole peanuts, and the increase was higher than that of intercropped maize and peanuts with board separation (Supplementary Table S3). This was consistent with the research finding that the soil N and NH_4_^+^-N contents of intercropped peanuts and intercropped cotton are higher than those of sole cropping, and the nutrient accumulation of peanut and cotton soil is significantly inhibited when the root system is separated (Xie et al., 2022). The root depths of maize and peanut differed in the intercropping system, thus improving soil nutrient cycling and uptake by niche complementation (Xue et al., 2016; Duchene et al., 2017; Li et al., 2020; Mahmoud et al., 2022). The results of this study also showed that NH_4_^+^-N changed significantly in the rhizosphere soil of intercropped maize, intercropped peanut, and the shared soil of intercropped maize and peanut (Supplementary Table S3). In maize intercropping, the existence of interspecific competition enhances the nitrogen fixation of peanut nodules, promotes nitrogen absorption and utilization, and activates the nitrogen cycle in the soil of the intercropping system (Xu et al., 2007; Shen et al., 2019; Li et al., 2021; Nasar et al., 2022). Generally, root interactions improve the soil nutrient cycle in the intercropping of maize and peanuts, especially in terms of the absorption and utilization of NH_4_^+^-N.

Soil enzyme activity is an important indicator of changes in the soil microbial activity (Li et al., 2019). A previous meta-analysis of a large body of literature found that intercropping significantly increased enzyme activity by an average of 13% (*p* < 0.001), and that the intercropping effects varied by enzyme class, main crop or plant type intercropped, and other experimental and environmental factors (Curtright and Tiemann, 2021). In this study we found that the enzyme activities of UE, and Pro increased significantly in intercropped maize (Supplementary Figures 2A,B), the enzyme activities of Pro, NR increased significantly in intercropped peanut (Supplementary Figures 2B,C), the enzyme activities of UE, Pro, and DHO increased in the shared soil of intercropped maize and peanut, and NR decreased (Supplementary Figure 2), compared with the shared soil of sole maize and sole peanut. This may be attributed to differences in the litter residues of different crops, different induced enzyme responses, and different intercropping effects (Malobane et al., 2020). We found that the increase in the UE, Pro, and DHO involved in the soil nitrogen cycle under the intercropping of maize and peanut was consistent with the results of other studies (Supplementary Figures 2A,B,D; Li et al., 2016b, 2022b; Tang et al., 2020, 2021). In addition, the increase in enzyme activity was generally higher than that in the root system separated treatment. Overall, these results suggest that interspecific root interactions increase soil enzyme activity and improve soil nutrient cycling capacity (Li et al., 2018), providing support for the first aim.

In the current study, the PCA showed that the root exudates in SM vs. IM, SP vs. IP, SMI vs. II, and SPI vs. II were significantly separated, indicating that the root interaction significantly affected the distribution of root exudates (Figures 2A,B). Plants secrete different types and amounts of compounds in the rhizosphere depending on plant size, photosynthetic activity, soil conditions, and species genotype (Mommer et al., 2016). Flavonoids are one of the most secondary metabolites secreted by plant roots and have various biological activities such as antioxidant and antimicrobial activities (Panche et al., 2016; Liu et al., 2021). It is reported, flavonoids play critical roles in the nodule nitrogen fixation and regulation of interplant and plant-microbe interactions (Weston and Mathesius, 2013). Compared with sole faba bean, the root of intercropped broad bean secreted the enrichment with flavonoids, for example, flavanols, isoflavone, chalcone and hesperetin, and the enhancement of faba bean nodulation (Liu et al., 2017). Compared with sole wheat, intercropping of wheat and faba bean secreted more flavonoids, and interspecific interaction changed the content and proportion of flavonoids in wheat root exudates (Liu et al., 2020). A total of 23 flavonoids and seven isoflavones compounds were detected in this study (Supplementary Table S4). In addition, the differences in flavonoids and isoflavones in SM vs. IM (10) and SMI vs. II (14) were greater than those in SP vs. IP (6) and SPI vs. II (13), respectively (Figure 3A), which exhibited more characteristics of peanut root exudates, further indicating the dominant role of peanuts in the distribution of flavonoids and isoflavones in II. Resource competition affects interspecific interactions, because maize is more competitive than peanuts for light, temperature, water, nutrients, etc., therefore, peanuts respond to neighboring maize by altering root chemistry and stimulating flavonoids biosynthesis and root secretion (Hazrati et al., 2021; Leoni et al., 2021). Five core root exudates shared by SMI vs. II and SPI vs. II were up regulated, including MW00129050, MW00138036, MW00131337, ZINC85832360, and MW00148166 (Figure 3B and Supplementary Table S4), identified as key differential root exudates affecting the process of root interaction. Specifically, the expression of cyanidin 3-sambubioside 5-glucoside (MW00138036) and 3-(3,4-Dimethoxyphenyl)-6-ethoxy-4-methylcoumarin was upregulated in intercropped maize; the expression of cyanidin 3-O-(6-Op-coumaroyl) glucoside-5-O-glucoside; shisonin was up-regulated in intercropped peanut (Supplementary Table S4). Among them, cyanidin 3-sambubioside 5-glucoside and cyanidin 3-O-(6-O-p-coumaroyl) glucoside-5-O-glucoside; Shisonin were involved in the anthocyanin synthesis, and it is the final product of the flavonoid metabolic pathway. The expression of nodule nitrogen-fixing genes *CHI*, *NODL4*, *ENODL2*, and *ENOD93* in the upstream stage of the flavonoid synthesis pathway was significantly upregulated (Hu et al., 2021; Jiang et al., 2022), which promoted the synthesis of flavonoids. This also explains the presence of cyanidin 3-sambubioside 5-glucoside and cyanidin 3-O-(6-Op-coumaroyl) glucoside-5-O-glucoside; shisonin were significantly up-regulated in the downstream stage of the anthocyanin synthesis pathway. This indicated that interspecific interactions regulated the types and contents of flavonoids excreted by the roots of maize and peanut.

Rhizosphere microbial communities benefit plants by increasing nutrient availability (Richardson et al., 2009; Jacoby et al., 2017), producing plant growth hormones (Nihorimbere et al., 2011; Carvalhais et al., 2019; Kyozuka et al., 2022), and defending against pathogens (Chen et al., 2014; Pang et al., 2022). A growing body of research indicates that interspecific root interactions affect the rhizosphere microbial community composition in intercropping systems (Wang et al., 2018; Jiang et al., 2022; Xie et al., 2022). This study showed that there were significant differences in the rhizosphere microbial community structure under intercropping, compared with sole cropping, especially in the SP vs. IP, and SPI vs. II (Figure 5B). Interspecific root interaction further caused differences in the bacterial community structure during intercropping. For instance, Li et al. (2022a) found that Actinobacteria and Proteobacteria were the dominant phyla, and that the relative abundance of Proteobacteria in intercropped maize rhizosphere soil increased significantly (Li et al., 2022a). Li et al. (2018) showed that the numbers of *Bacillus*, *Brevibacillus brevis*, and *Paenibacillus* mainly increased in the maize rhizosphere. *Burkholderia*, *Pseudomonas*, and *Rhizobium* mainly increased in the peanut rhizosphere (Li et al., 2018). Some bacterial changes were consistent with our study, this phenomenon may be due to secondary metabolites altering the abundance of bacteria. Most notably, the abundance of the dominant bacterial genus *Bradyrhizobium* increased in the intercropped maize, intercropped peanut, and the shared soil of intercropped maize and peanut (Figure 5A and Supplementary Table S5), which was consistent with the previous study (Li et al., 2018; Pang et al., 2021). A recent study suggested that *Bradyrhizobium* promotes nodulation and nitrogen fixation in peanuts during intercropping (Solanki et al., 2019; Pang et al., 2021; Chen et al., 2022). Hence, it may play an important role in promoting nitrogen uptake and transport in the intercropping of maize and peanut. Our next step was to perform isolation and verification. Alterations in the composition of the rhizosphere microbial community were due to fertilization (Guo et al., 2020), crop type (Prommer et al., 2020), and above-ground processes related to crop diversity, such as microclimate changes related to plant canopy cover (Delgado-Baquerizo et al., 2018), root exudates (Mommer et al., 2016), and soil environmental factors (Stefan et al., 2021). Root exudates are important factors that affect the rhizosphere microbial communities (Wang et al., 2022).To further clarify our second aim the relationship between flavonoids root exudates and soil bacterial community composition in intercropping systems, spearman’s correlation analysis revealed significant correlations between specific root exudates and bacterial community composition. Among these, cyanidin 3-sambubioside 5-glucosideand cyanidin 3-O-(6-Op-coumaroyl) glucoside-5-O-glucoside; shisonin had frequent significantly positive correlations with bacterial communities; the greatest correlation was with *Bradyrhizobium* (Figure 6A and Supplementary Table S6), indicating that some root exudates secreted by the root changed the bacterial community composition, while, some bacterial metabolic pathways also changed to adapt to environmental stress (Zhao et al., 2022b). These results showed that root exudates could be used to assess the adaptations of soil microbial communities to interspecific interactions at the molecular level. Furthermore, in this study, Pro, NH_4_^+^-N and UE had significantly positive correlation with g_*Bradyrhizobium* and core root exudates (Figure 6A and Supplementary Table S7), suggesting that root interactions improve plant resource availability by changing root exudates and rhizosphere bacterial. In conclusion, root interactions cause differences and changes in root exudates, regulate bacterial community composition, alter soil physicochemical properties, and promote plant growth and development. To investigate the potentially important predictors of plant yield, we conducted random forest modelling with the nitrogen accumulation per plant of maize and peanut, soil nutrients (TN: soil total N; NH_4_^+^-N: soil ammonium N), soil enzyme activity (UE: soil urease; NR: soil nitrate reductase; Pro: soil protease; DHO: soil dehydrogenase), core root exudates in the shared soil of intercropped maize and peanut, and *Bradyrhizobium*. Thus, we found some evidence of our third aim. Soil UE was the most important predictor of plant yield (Figure 6B). This was followed by plant N, soil physicochemical properties and root exudates (Figure 6B). Overall, these findings suggest that intercropping interspecific interactions could change rhizosphere soil physicochemical properties, reshape rhizosphere soil bacterial communities, and enrich nitrogen-fixing bacteria *Bradyrhizobium,* which promote nitrogen uptake and yield in intercropped maize.

## Conclusion

Our study showed that intercropped maize yields and nitrogen accumulation significantly increased, compared to sole maize. Therefore, we went on to explore how root interactions in the belowground of maize and peanut regulate yield increase. Our results revealed that the release of flavonoids increased, and cyanidin 3-sambubioside 5-glucoside and cyanidin 3-O-(6-Op- coumaroyl) glucoside-5-O-glucoside; shisonin were up-regulated in the shared soil of intercropped maize and peanut. Correlation analysis showed that flavonoids were significantly positively correlated with the content of NH_4_^+^-N, and the activity of UE, Pro, DHO and nitrogen-fixing bacteria *Bradyrhizobium.* Random forest analysis further demonstrated that interspecific interactions modulated flavonoids secretion, affected soil nitrogen content and enzymatic activity, and reassemble rhizosphere bacterial community. Thereby, promoting nitrogen fixation in peanut nodules and nitrogen absorption in maize and achieving high yield in the intercropped maize. This study enhances our understanding of the ecological role of root exudates and rhizosphere soil microbes in the intercropping of maize and peanut.

## Data availability statement

The datasets presented in this study can be found in online repositories. The names of the repository/repositories and accession number(s) can be found at: https://www.ncbi.nlm.nih.gov/, PRJNA833565.

## Author contributions

HY, XZ, and QD designed this study. QD conducted the data analysis and wrote the manuscript. DZ, YY, ZL, PJ, YL, and PS carried out the field experiments. XS, HZ, and CZ helped in data analysis. XW, CJ, XL, FG, ZZ, and SW revised the manuscript. All authors contributed to the article and approved the submitted version.

## Funding

This research was funded by the Joint Funds of the National Natural Science Foundation of China (U21A20217) and the China Agricultural Research System (CARS-13).

## Conflict of interest

The authors declare that the research was conducted in the absence of any commercial or financial relationships that could be construed as a potential conflict of interest.

## Publisher’s note

All claims expressed in this article are solely those of the authors and do not necessarily represent those of their affiliated organizations, or those of the publisher, the editors and the reviewers. Any product that may be evaluated in this article, or claim that may be made by its manufacturer, is not guaranteed or endorsed by the publisher.

## Abbreviations

SM: Sole maize
IDM: Intercropped maize with board separation
IM: Intercropped maize
SP: Sole peanut
IDP: Intercropped peanut with board separation
IP: Intercropped peanut
SMI: The shared soil of sole maize
SPI: The shared soil of sole peanut
II: The shared soil of intercropped maize and peanut
TN: Total nitrogen
NH_4_^+^-N: Ammonium nitrogen
UE: Urease
Pro: Protease
NR: Nitrate reductase
DHO: Dehydrogenase
