# Supplementary material for: Maize and peanut intercropping improves the nitrogen accumulation and yield per plant of maize by promoting the secretion of flavonoids and abundance of Bradyrhizobium in rhizosphere
Source: Front Plant Sci. 2022 Aug 4;13:957336. doi: 10.3389/fpls.2022.957336 (PMC9386453; doi:10.3389/fpls.2022.957336)
Supplement: Supplementary file 1 [file Data_Sheet_1.docx]

***Supplementary Material***

# Supplementary Data

**Text S1**

A total amount of 1μg DNA per sample was used as input material for the DNA sample preparations. Sequencing libraries were generated using NEBNext® Ultra™ DNA Library Prep Kit for Illumina (NEB, USA) following manufacturer’s recommendations and index codes were added to attribute sequences to each sample. Briefly, the DNA sample was fragmented by sonication to a size of 350bp, then DNA fragments were end-polished, A-tailed, and ligated with the full-length adaptor for Illumina sequencing with further PCR amplification. The PCR primer sequences 799F (5’-AACMGGATTAGATACCCKG-3’) (Chelius and Triplett, 2001) and 1193R (5’- ACGTCATCCCCACCTTCC-3’) (Bodenhausen et al., 2013) were used during the first-round PCR to amplify the V5–V7 regions of 16S rRNA genes. The PCR reactions were assembled in a laminar flow and amplified using protocol; 95 °C for 3 min, 25 cycles of 95 °C for 30 s, 55°C for 30 s, 72 °C for 30 s, and extension at 72 °C for 5 min (Kaushal et al., 2021). At last, PCR products were purified (AMPure XP system) and libraries were analyzed for size distribution by Agilent2100 Bioanalyzer and quantified using real time PCR. The clustering of the index-coded samples was performed on a cBot Cluster Generation System according to the manufacturer’s instructions. After cluster generation, the library preparations were sequenced on an Illumina HiSeq platform and paired-end reads were generated. The Raw Data obtained from the Illumina HiSeq sequencing platform using Readfq (V8, https://github.com/cjfields/readfq) was conducted to acquire the clean data for subsequent analysis with QIME v1.91 software (Lawley and Tannock, 2017).

# Supplementary Figures and Tables

## Supplementary Figures


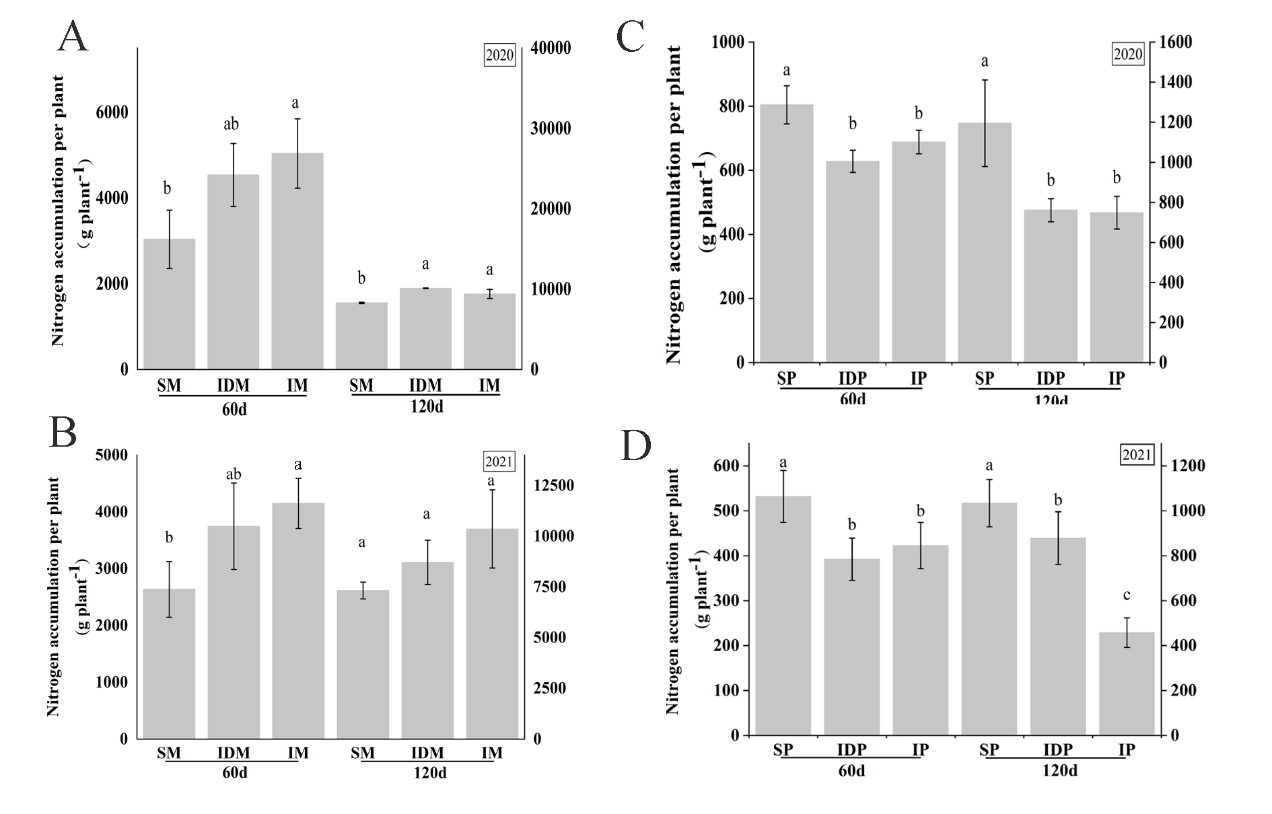


**Supplementary Figure 1.** Nitrogen accumulation per plant in maize and peanut under different planting patterns. (A, B) Nitrogen accumulation per maize plant in 2020 and 2021. (C, D) Nitrogen accumulation per peanut plant in 2020 and 2021. SM: sole maize, IDM: intercropped maize with board separation, IM: intercropped maize, SP: sole peanut, IDP: intercropped peanut with board separation, IP: intercropped peanut. Different lowercase letters represent significant difference level *p* =0.05 using Duncan’s test, as follows.


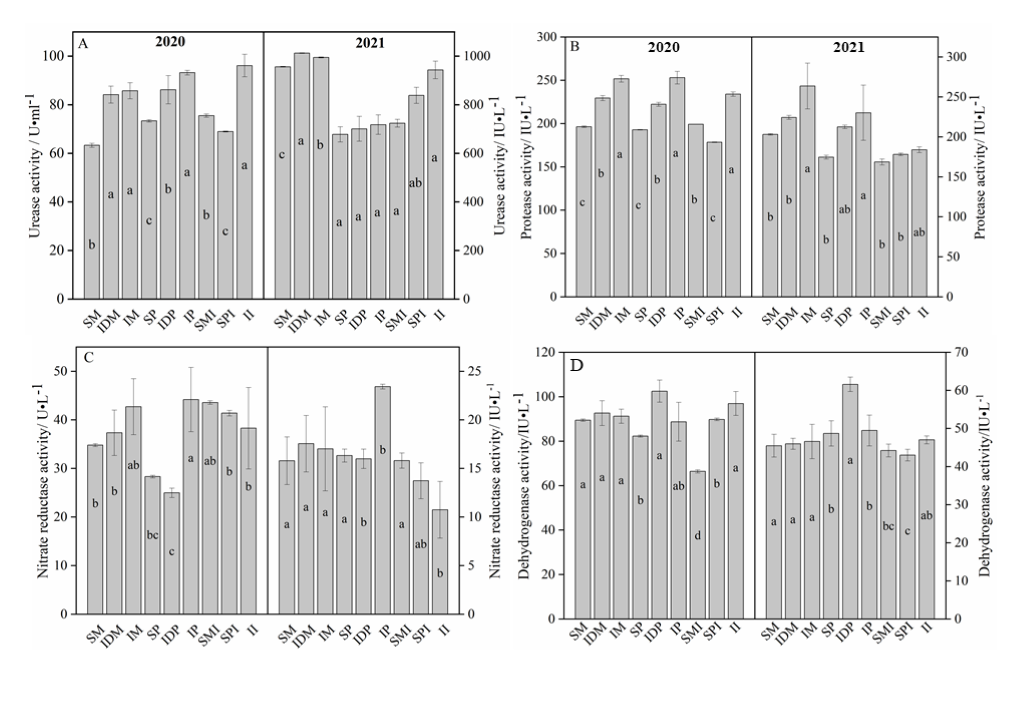


**Supplementary Figure 2.** Soli enzyme activities in maize and peanut rhizosphere soil under different planting patterns. SM: sole maize, IM: intercropped maize, SP: sole peanut, IP: intercropped peanut, SMI: the shared soil of sole maize, SPI: the shared soil of sole peanut, II: the shared soil of intercropped maize and peanut.


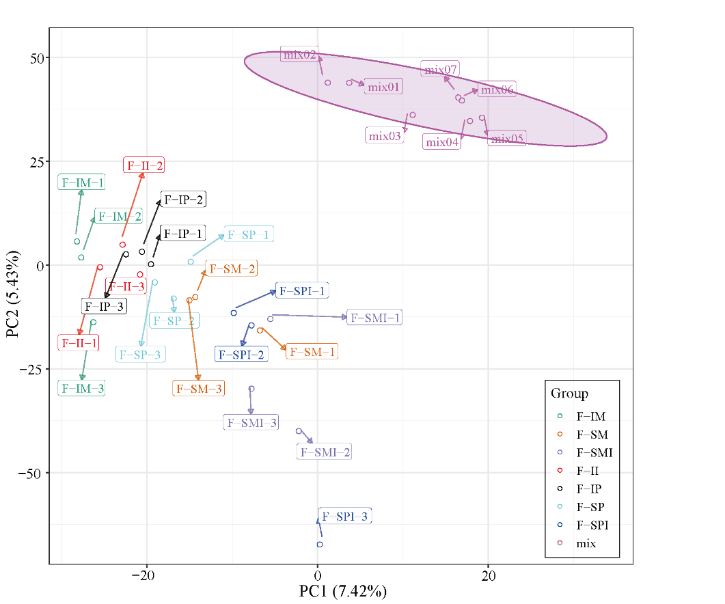


**Supplementary Figure 3.** PCA of mass spectrometry data of each group of samples and quality control samples. The X: the first principal component, the Y: represents the second principal component, mix represents the quality control sample. SM: sole maize, IM: intercropped maize, SP: sole peanut, IP: intercropped peanut, SMI: the shared soil of sole maize, SPI: the shared soil of sole peanut, II: the shared soil of intercropped maize and peanut.


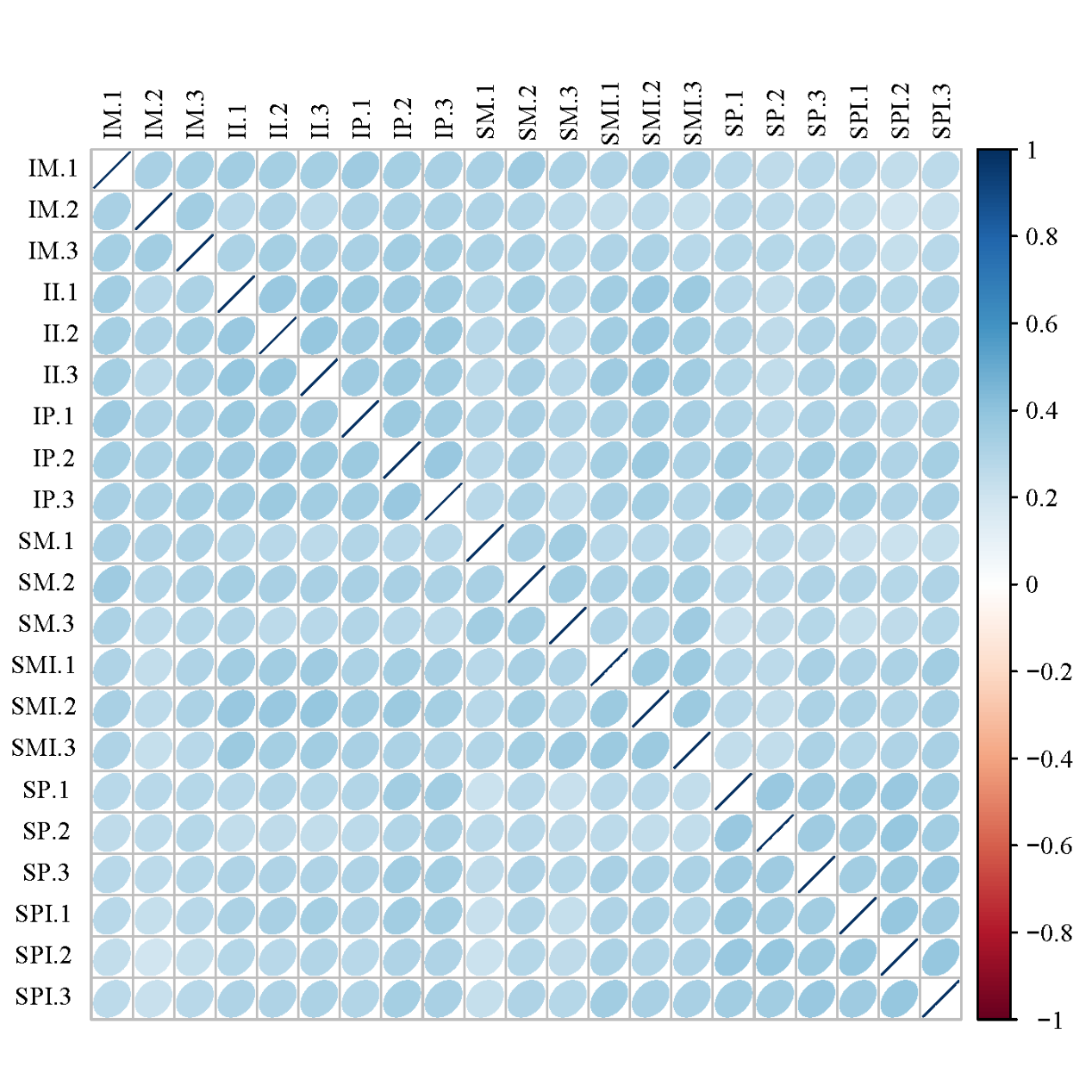


**Supplementary Figure 4.** Correlation heatmap of gene abundance for each sample. SM: sole maize, IM: intercropped maize, SP: sole peanut, IP: intercropped peanut, SMI: the shared soil of sole maize, SPI: the shared soil of sole peanut, II: the shared soil of intercropped maize and peanut.

## Supplementary Tables

**Table S1** The amount of fertilizer applied per planting patterns (g box^-1^)

| **Planting patterns** | **Peanut** | | |  | **Maize** | | |
| --- | --- | --- | --- | --- | --- | --- | --- |
|  | **N** | **P_2_O_5_** | **K_2_O** |  | **N** | **P_2_O_5_** | **K_2_O** |
| **SM/SP** | 13.5 | 90 | 18 |  | 27 | 12.75 | 14.25 |
| **ID** | 6.75 | 45 | 9 |  | 13.5 | 6.375 | 7.125 |
| **IMP** | 6.75 | 45 | 9 |  | 13.5 | 6.375 | 7.125 |

SM: sole peanut, SP: sole peanut, ID: intercropping of maize and peanut with board separation, IMP: intercropping of maize and peanut.

**Table S2** Nitrogen accumulation in various organs of maize and peanut under different planting patterns (g plant^-1^)

| **Year** | **Planting** | **60d** | | |  | **120d** | | | |
| --- | --- | --- | --- | --- | --- | --- | --- | --- | --- |
|  | **patterns** | **Root** | **Stem** | **Leaf** |  | **Root** | **Stem** | **Leaf** | **Grain/Pod** |
| **2020** | **SM** | 455.44±61.75a | 908.39±210.31c | 1675.02±497.04a |  | 527.24±126.06b | 989.28±41.78b | 1415.04±92.81b | 5365.57±231.02b |
|  | **IDM** | 595.19±50.14a | 1806.55±108.07b | 2138.55±661.18a |  | 655.46±73.30b | 991.80±37.97b | 1161.75±65.06b | 7299.41±66.43a |
|  | **IM** | 562.51±80.81a | 2740.36±68.81a | 1737.93±804.90a |  | 1208.22±96.19a | 1531.52±181.58a | 2013.71±253.99a | 4649.58±762.62b |
| **2021** | **SM** | 442.56±80.07a | 694.64±274.65b | 1497.67±475.69a |  | 446.87±204.09b | 939.20±68.22a | 1630.58±279.28a | 4306.31±467.06a |
|  | **IDM** | 593.48±161.91a | 1565.38±127.10a | 1589.69±653.17a |  | 413.18±171.57b | 948.93±261.80a | 1233.94±225.49b | 6121.48±763.5a |
|  | **IM** | 710.13±181.23a | 2014.36±306.11a | 1424.82±447.78a |  | 1125.82±468.13a | 1744.23±665.71a | 2520.55±415.53b | 4973.25±1465.78a |
| **Year** | | 0.514 | 0.006 | 0.360 |  | 0.332 | 0.824 | 0.093 | 0.179 |
| **Planting patterns** | | 0.090 | 0.000 | 0.521 |  | 0.002 | 0.012 | 0.000 | 0.006 |
| **Y×P** | | 0.557 | 0.175 | 0.854 |  | 0.853 | 0.788 | 0.482 | 0.345 |
| **2020** | **SP** | 18.44±9.49a | 256.25±48.62a | 529.49±212.67a |  | 51.02±8.21a | 255.45±53.57a | 171.87±42.02b | 716.83±257.53a |
|  | **IDP** | 9.74±0.62a | 211.87±70.78a | 406.45±139.72a |  | 44.12±9.38a | 107.28±25.93b | 251.55±41.53a | 358.38±78.60ab |
|  | **IP** | 16.92±4.54a | 243.12±1.62a | 411.76±37.13a |  | 18.30±3.23c | 110.38±40.55b | 143.37±12.71bc | 476.25±61.95c |
| **2021** | **SP** | 10.6±3.11a | 199.14±45.38a | 322.23±35.80a |  | 9.11±3.52a | 168.34±52.38a | 132.92±36.89a | 723.30±72.98a |
|  | **IDP** | 8.92±2.87a | 146.62±22.01b | 236.91±34.61bc |  | 10.19±10.98a | 146.48±34.98ab | 123.78±21.68a | 597.75±87.73b |
|  | **IP** | 9.18±1.75a | 170.36±29.69ab | 243.14±27.89b |  | 7.69±3.42a | 77.37±10.37c | 87.26±17.44b | 285.43±49.34c |
| **Year** | | 0.000 | 0.000 | 0.000 |  | 0.009 | 0.000 | 0.001 | 0.299 |
| **Planting patterns** | | 0.111 | 0.000 | 0.026 |  | 0.000 | 0.000 | 0.000 | 0.000 |
| **Y×P** | | 0.138 | 0.932 | 0.549 |  | 0.000 | 0.000 | 0.000 | 0.001 |

SM: sole maize, IDM: intercropped maize with board separation, IM: intercropped maize, SP: sole peanut, IDP: intercropped peanut with board separation, IP: intercropped peanut.

**Table S3** Rhizosphere soil nitrogen content of maize and peanut under different planting patterns

| **Year** | **Planting patterns** | **60d** | |  | **120d** | |
| --- | --- | --- | --- | --- | --- | --- |
|  |  | **TN (mg g-1)** | **NH_4_^+^-N (mg g-1)** |  | **TN (mg g-1)** | **NH_4_^+^-N (mg g-1)** |
| **2020** | **SM** | 0.96±0.26a | 0.049±0.0024b |  | 1.31±0.12a | 0.047±0.0042b |
|  | **IDM** | 1.47±0.22a | 0.037±0.0160b |  | 0.77±0.34b | 0.066±0.0080ab |
|  | **IM** | 1.51±0.30a | 0.106±0.0115a |  | 1.49±0.15a | 0.128±0.0687a |
|  | **SP** | 0.81±0.54a | 0.056±0.0039b |  | 1.27±0.15b | 0.052±0.0005a |
|  | **IDP** | 1.31±0.40a | 0.054±0.0052b |  | 0.97±0.53b | 0.064±0.0025a |
|  | **IP** | 1.61±0.13a | 0.093±0.0023a |  | 1.75±0.18a | 0.085±0.0025a |
|  | **SMI** | 1.05±0.18a | 0.058±0.0005b |  | 1.52±0.5a | 0.06±0.0141b |
|  | **SPI** | 1.17±0.17a | 0.059±0.0007b |  | 1.34±0.24a | 0.062±0.0112b |
|  | **II** | 1.93±0.21a | 0.101±0.0080a |  | 1.54±0.03a | 0.137±0.0445a |
| **2021** | **SM** | 2.89±0.08a | 0.060±0.0065b |  | 2.51±0.18a | 0.034±0.0047b |
|  | **IDM** | 2.67±0.21a | 0.042±0.0036c |  | 2.75±0.35a | 0.045±0.0033b |
|  | **IM** | 3.02±0.54a | 0.147±0.0054a |  | 2.85±0.32a | 0.096±0.0029a |
|  | **SP** | 2.22±0.27a | 0.028±0.0117c |  | 2.46±0.27a | 0.040±0.0072b |
|  | **IDP** | 2.58±0.30a | 0.074±0.0356b |  | 2.38±0.47a | 0.041±0.0060b |
|  | **IP** | 2.86±0.68a | 0.122±0.0097a |  | 2.62±0.05a | 0.078±0.0247a |
|  | **SMI** | 1.05±0.18a | 0.064±0.0027b |  | 2.34±0.58a | 0.037±0.0146b |
|  | **SPI** | 1.17±0.17a | 0.054±0.0053c |  | 2.12±0.15a | 0.046±0.0108ab |
|  | **II** | 1.93±0.21a | 0.087±0.0012a |  | 2.49±0.08a | 0.063±0.0022a |
| **Year** | | 0.000 | 0.058 |  | 0.000 | 0.001 |
| **Planting patterns** | | 0.018 | 0.000 |  | 0.228 | 0.000 |
| **Y×P** | | 0.503 | 0.002 |  | 0.184 | 0.546 |

SM: sole maize, IDM: intercropped maize with board separation, IM: intercropped maize, SP: sole peanut, IDP: intercropped peanut with board separation, IP: intercropped peanut, SMI: the shared soil of sole maize, SPI: the shared soil of sole peanut, II: the shared soil of intercropped maize and peanut, as follows.

**Table S5** The relative abundance of bacterial communities at the phylum and genus levels (%)

|  | **Bacterial** | **The relative abundance in bacterial (%)** | | | | | | | |
| --- | --- | --- | --- | --- | --- | --- | --- | --- | --- |
|  |  | **IM** | **SM** | **IP** | **SP** | **SMI** | **SPI** | **II** | **Mean** |
| **phylum** | p_Proteobacteria | 33.12% | 32.12% | 31.80% | 34.71% | 30.91% | 33.91% | 31.84% | 32.63% |
|  | p_Actinobacteria | 19.52% | 18.45% | 16.91% | 17.43% | 16.31% | 15.07% | 15.58% | 17.04% |
|  | p_Acidobacteria | 14.90% | 15.48% | 16.56% | 13.91% | 17.25% | 15.45% | 17.70% | 15.89% |
|  | p_Gemmatimonadetes | 4.07% | 4.52% | 4.92% | 5.81% | 4.73% | 5.89% | 4.62% | 4.94% |
|  | p_Verrucomicrobia | 1.79% | 2.20% | 2.01% | 1.93% | 2.41% | 2.31% | 2.13% | 2.11% |
|  | p_Chloroflexi | 1.90% | 2.13% | 1.90% | 1.96% | 2.03% | 2.05% | 1.93% | 1.99% |
|  | p_Bacteroidetes | 1.42% | 1.35% | 1.56% | 1.45% | 1.32% | 1.36% | 1.28% | 1.39% |
|  | p_Planctomycetes | 0.96% | 1.00% | 1.02% | 0.86% | 1.03% | 0.89% | 1.01% | 0.97% |
|  | p_Thaumarchaeota | 0.53% | 0.62% | 0.66% | 0.68% | 0.66% | 0.74% | 0.61% | 0.64% |
|  | p_Nitrospirae | 0.90% | 1.00% | 0.97% | 0.80% | 0.91% | 0.81% | 0.90% | 0.90% |
|  | Others | 20.90% | 21.15% | 21.70% | 20.45% | 22.43% | 21.53% | 22.40% | 21.51% |
| **genus** | g_*Sphingomonas* | 2.61% | 2.48% | 2.60% | 6.04% | 2.10% | 5.04% | 2.22% | 3.30% |
|  | g_*Rhodanobacter* | 0.69% | 0.97% | 0.28% | 0.41% | 0.19% | 0.21% | 0.19% | 0.42% |
|  | g_*Bradyrhizobium* | 1.59% | 1.24% | 1.51% | 1.46% | 1.27% | 1.29% | 1.35% | 1.39% |
|  | g_*Pseudolabrys* | 0.87% | 0.85% | 0.86% | 0.82% | 0.75% | 0.73% | 0.83% | 0.82% |
|  | g_*Haliangium* | 0.71% | 0.55% | 0.79% | 0.61% | 0.82% | 0.71% | 0.84% | 0.72% |
|  | g_*Streptomyces* | 1.40% | 1.65% | 0.92% | 0.85% | 1.20% | 0.68% | 0.90% | 1.09% |
|  | g_*Nocardioides* | 1.10% | 0.89% | 0.92% | 1.00% | 0.76% | 0.77% | 0.73% | 0.88% |
|  | g_*Candidatus Solibacter* | 1.94% | 2.04% | 2.08% | 1.49% | 2.18% | 1.66% | 2.31% | 1.96% |
|  | g_*Candidatus Koribacter* | 0.93% | 0.90% | 1.10% | 0.85% | 1.11% | 0.96% | 1.19% | 1.01% |
|  | g_*Gemmatirosa* | 1.28% | 1.41% | 1.56% | 1.85% | 1.45% | 1.84% | 1.43% | 1.54% |
|  | Others | 86.88% | 87.01% | 87.39% | 84.63% | 88.17% | 86.13% | 88.01% | 86.89% |

Bodenhausen, N., Horton, M.W., Bergelson, J., 2013. Bacterial communities associated with the leaves and the roots of Arabidopsis thaliana. PLoS One 8, e56329.

Chelius, M.K., Triplett, E.W., 2001. The Diversity of Archaea and Bacteria in Association with the Roots of Zea mays L. Microb Ecol 41, 252-263.

Kaushal, R., Peng, L., Singh, S.K., Zhang, M., Zhang, X., Vilchez, J.I., Wang, Z., He, D., Yang, Y., Lv, S., Xu, Z., Morcillo, R.J.L., Wang, W., Huang, W., Pare, P.W., Song, C.P., Zhu, J.K., Liu, R., Zhong, W., Ma, P., Zhang, H., 2021. Dicer-like proteins influence Arabidopsis root microbiota independent of RNA-directed DNA methylation. Microbiome 9, 57.

Lawley, B., Tannock, G.W., 2017. Analysis of 16S rRNA Gene Amplicon Sequences Using the QIIME Software Package. Methods in molecular biology (Clifton, N.J.) 1537, 153-163.
